# Susceptibility of Red Mason Bee Larvae to Bacterial Threats Due to Microbiome Exchange with Imported Pollen Provisions

**DOI:** 10.3390/insects11060373

**Published:** 2020-06-15

**Authors:** Anna Voulgari-Kokota, Ingolf Steffan-Dewenter, Alexander Keller

**Affiliations:** 1 Department of Bioinformatics, Biocenter, University of Würzburg, Am Hubland, 97074 Würzburg, Germany; a.voulgari.kokota@rug.nl; 2 Center for Computational and Theoretical Biology, Biocenter, University of Würzburg, Hubland Nord, 97074 Würzburg, Germany; 3 Current Address: Faculty of Science and Engineering, Groningen Institute for Evolutionary Life Sciences, Nijenborgh 7, 9747 AG Groningen, The Netherlands; 4 Department of Animal Ecology and Tropical Biology, Biocenter, University of Würzburg, Am Hubland, 97074 Würzburg, Germany; ingolf.steffan-dewenter@uni-wuerzburg.de

**Keywords:** *Osmia bicornis*, solitary bee, bacterial transmission, microbiome, pollen provisions, pathogen, secondary invader, *Paenibacillus*, *Bacillus*, *Sporosarcina*

## Abstract

Solitary bees are subject to a variety of pressures that cause severe population declines. Currently, habitat loss, temperature shifts, agrochemical exposure, and new parasites are identified as major threats. However, knowledge about detrimental bacteria is scarce, although they may disturb natural microbiomes, disturb nest environments, or harm the larvae directly. To address this gap, we investigated 12 *Osmia bicornis* nests with deceased larvae and 31 nests with healthy larvae from the same localities in a 16S ribosomal RNA (rRNA) gene metabarcoding study. We sampled larvae, pollen provisions, and nest material and then contrasted bacterial community composition and diversity in healthy and deceased nests. Microbiomes of pollen provisions and larvae showed similarities for healthy larvae, whilst this was not the case for deceased individuals. We identified three bacterial taxa assigned to *Paenibacillus* sp. (closely related to *P. pabuli/amylolyticus/xylanexedens*), *Sporosarcina* sp., and *Bacillus* sp. as indicative for bacterial communities of deceased larvae, as well as *Lactobacillus* for corresponding pollen provisions. Furthermore, we performed a provisioning experiment, where we fed larvae with untreated and sterilized pollens, as well as sterilized pollens inoculated with a *Bacillus* sp. isolate from a deceased larva. Untreated larval microbiomes were consistent with that of the pollen provided. Sterilized pollen alone did not lead to acute mortality, while no microbiome was recoverable from the larvae. In the inoculation treatment, we observed that larval microbiomes were dominated by the seeded bacterium, which resulted in enhanced mortality. These results support that larval microbiomes are strongly determined by the pollen provisions. Further, they underline the need for further investigation of the impact of detrimental bacterial acquired via pollens and potential buffering by a diverse pollen provision microbiome in solitary bees.

## 1. Introduction

Solitary bees are providers of invaluable ecological services [1,2], and increasing demand for pollination services results in their health being at the center of agro-economic research [3]. The decline in solitary bee populations raises concerns regarding the environmental pressures they face [4,5,6]. Most studies on possible threats against bees focused on perils caused by increasing land-use intensity, landscape fragmentation, parasites, or climate change [7,8,9]. Studies on honey bees revealed bacterial agents which can cause acute mortality in the hive [10]. *Paenibacillus larvae* [11] and *Melissococcus plutonius* [12] are widely accepted as the main causative pathogens for American and European Foulbrood in larvae, respectively. Furthermore, the honey bee pathogens *Spiroplasma apis* and *Spiroplasma melliferum* [13,14] can reduce adult bee longevity. Studies on regional scales report that pathogens also affect the abundance of bumble bees. Common reported pathogens of *Bombus* are trypanosomes and microsporidia, which are transmitted horizontally between bumble bee colonies and vertically within colonies [15,16]. In particular, the bumblebee *Bombus terrestris* and its parasite *Crithidia bombi* were studied extensively to determine the role of brood in disease transmission [17]. Likewise to honey bees, *Spiroplasma* spp. were also identified as bacterial pathogens for bumble bees [10,18]. Considering solitary bees, knowledge about their natural and healthy microbiomes advanced in the recent years [19,20,21,22,23,24]. Studies investigating potential microbial pathogens, however, focused on infections that are common between honey bees or bumble bees and solitary bees [25,26,27]. While studies investigated non-lethal endosymbionts in solitary bees, e.g., *Wolbachia* [28], dedicated studies screening for potential bacterial pathogens and other harmful bacteria in solitary bees specifically are currently lacking.

In this study, we investigated the bacterial communities associated with deceased *Osmia bicornis* larvae and contrasted these with healthy larvae sampled from the same locations. *O. bicornis* are widespread and abundant in Europe, and the females are generalists and effective pollinators, transferring pollen from numerous plant species into their nests [29]. They use soil to separate individual chambers within a reed nest, each containing a single egg. Individual nest chambers are immediately closed, and larvae are afterwards not actively nursed, thereby preventing continuous vertical transmission of bacteria between mothers or other nest-mates and offspring [30]. Horizontal transmission of microbes hitch-hiking collected pollen provisions and nest materials, however, is regarded as a central bacterial acquisition route [22,30,31,32]. Seasonal and geographic segregation, as well as differences in foraging preferences from other co-existing bee species might determine which microbes are imported [22]. Such horizontally and environmentally obtained microbes might cover important functions in health and nutrition similar to those covered by vertically transmitted ones observed in close association with corbiculate bees [32].

*Osmia bicornis* nests harbor a variety and large proportion of environmentally introduced bacteria [19,23,30], making this bee species an ideal candidate for testing susceptibility to environmental and opportunistic bacterial infections. We collected nests containing deceased larvae and well-developed, healthy larvae lacking visible parasites or molds from the same locations. Larvae, their pollen provision, and the soil pellet used to seal and separate nest chambers were screened by 16S ribosomal RNA (rRNA) gene metabarcoding. We tested the hypothesis that healthy larvae and their respective nest materials (pollen provisions and chamber separators) share similarly composed bacterial communities, while the deceased ones show a prevalence of potentially pathogenic bacteria and a shift in their bacterial microbiome structure.

We further experimentally tested the hypothesis that the microbiome of the pollen provision impacts the larval microbiome. This was done to assess the roles that imported food provisions might play with respect to bacterial transmission and, thus, susceptibility to bacterial threats and successful development of the larvae. For this, we reared another 31 healthy larvae from the same nests and divided them into three groups undergoing different treatments. The first two groups were either fed with untreated pollen or with sterilized pollen. The last group fed on pollen, which was firstly sterilized and then inoculated with a *Bacillus* strain previously isolated from one of the deceased larvae. These artificially provisioned larvae were checked for survival and screened by 16S rRNA gene metabarcoding.

## 2. Materials and Methods

### 2.1. Sampling and Provisioning Experiment

Sampling took place in northern Bavaria, Germany. Artificial trap nests were established on the sites in April 2016 and thereafter checked regularly. Occupied nests were collected in the time frame from 25 May 2016 to 30 June 2016. Each trap nest consisted of two 20-cm-long polyvinyl chloride (PVC) tubes containing 30–50 reed sticks, which were placed on the top of 1-m-high wooden sticks. Pairwise minimum distance between sampling sites was 3.3 km, and pairwise maximum distance was 63.77 km. The sampling sites were principally agricultural land and semi-natural vegetation. The setup was part of a larger sampling project [23] and, for this study, we specifically targeted and selected by visual inspection the *O. bicornis* nests occupied with deceased larvae. We also excluded nests with deceased larvae apparently affected by molding cell interiors or the main parasites (dipterans, wasps, beetles, and mites) by visual inspection. In total, we collected 12 nests from eight localities that fit these criteria. Another 31 nests with healthy larvae were collected from the same sites as control samples. Further sample details are provided as Supplementary Table S1. Samples were obtained with permission by the Government of Unterfranken with reference number RUF-55.1.2-8646.11-1-16-6. Occupied nests were transferred into the lab, and they were opened horizontally in order to not disrupt the nest chambers.

For metabarcoding, we removed from each of the 12 nests with deceased larvae the rearmost deceased female larva. Moreover, we separately removed its respective remaining pollen provision and the soil separator of its respective nest chamber, resulting in a total of 36 samples (12 larvae, 12 pollen provisions, and 12 soil separators). Following the same strategy, we retrieved the rearmost female larva from each of the other 31 nests with only healthy larvae, as well as their pollen provisions and the soil from the nest chamber (93 samples). We selected the rearmost healthy or deceased larvae to investigate only females in this study, given that female cells are provided with more pollens and are located to the rear of the whole nest [33]. All 129 samples were transferred into sterile 1.5-mL tubes with the use of sterile tweezers. These samples were stored at −25 °C until further processing.

In addition, we retrieved one more larva from each of the 31 healthy nests in order to rear and manipulate them under controlled conditions. For this, the smallest remaining (therefore, probably also female larvae) larvae were directly transferred into sterile 48-well plates [34]. These 31 larvae were divided into three groups, and each group underwent a different treatment in the same closed microbiological safety cabinet. Eleven of the larvae were placed on pollen clumps retrieved from one *O. bicornis* nest without any further treatment (group A). Some of the untreated pollen was kept aside and included in bacterial metabarcoding as a “day 0 pollen control”. Furthermore, 10 larvae (group B) were placed on sterilized pollen clumps. The pollen provisions were sterilized by freeze-drying, soaking in 100 mg/L oxytetracycline for 24 h, and drying under ultraviolet (UV) overnight; then they were mixed with 10 µL of double-distilled water (ddH_2_O) and administered by pipetting [31]. This was also done to exclude the possibility that manipulation via sterilization would alter feeding behavior by artificially changing the microbial structure [35,36]. Finally, another 10 larvae were placed on pollen provisions which were firstly sterilized in the same way and then inoculated with a bacterial solution which contained a *Bacillus* sp. strain (group C). The *Bacillus* strain was previously isolated from one deceased larva (Sample ID: RP00401ML, see provided Supplementary Table S1) and cultivated on R2 agar (Merck, Darmstadt, Germany) at 37 °C for three days. An aliquot of the culture was series-diluted to 0.1 optical density at 600 nm (OD_600_), of which 1 μL was used for inoculating sterilized pollen and thoroughly mixed with a further 9 µL of ddH_2_O. This mixture was split equally and then provided to the 10 larvae of group C by pipetting. This was equal to approximately 1000–5000 bacterial cells per sample, with this range provided as we cannot be certain about the final proportion of living cells. After the larvae of all groups fed for five days, we transferred them into sterile 1.5-mL tubes and stored them at −25 °C. Six out of the 10 larvae of group C died on the fourth day of the experiment and were therefore directly stored at −25 °C one day earlier than the rest.

### 2.2. 16S rRNA Gene Metabarcoding

After sampling and concluding the provisioning experiment, we included all field-sampled larvae (12 deceased and 31 healthy), their respective pollen provisions and nest material, and the 31 experimental larvae plus day 0 pollen control in all downstream procedures (161 samples in total). All larvae were carefully rinsed with ddH_2_O-based 0.02% ethylene diaminetetraacetic acid (EDTA)/phosphate-buffered saline (PBS) prior to the DNA extraction. Genomic DNA from each of the samples was isolated using the Macherey-Nagel Nucleospin (Düren, Germany) kits for Food and Soil [37], following the protocol aiming at the better representation of bacteria with hard cell walls.

PCR amplification of the V4 region of the 16S ribosomal DNA (16S rRNA gene) followed a dual-indexing strategy [38]. The sequences of the primers used were AATGATACGGCGACCACCGAGATCTACAC (8bp-i5 index) ATGGTAATTGTGTGCCAGCMGCCGCGGTAA and CAAGCAGAAGACGGCATACGAGAT (8bp-i7 index) AGTCAGTCAGCCGGACTACHVGGGTWTCTAAT. To reduce random effects, PCR reactions were conducted in triplicate with 1 µL of template DNA in each reaction. Each reaction was prepared with New England Biolabs (UK) PCR Master Mix, along with the two indexed primers in a unique combination for each sample and an appropriate quantity of PCR-grade dH_2_O. PCR conditions were adjusted according to the primer guidelines [38]. After the end of the reaction, triplicates were combined per sample, and successful amplification was checked through gel electrophoresis on a 1% agarose gel.

DNA concentration was normalized between samples using the Invitrogen SequalPrep Plate Normalization Kit (ThermoFisher Scientific, Life Technologies, Carlsbad, CA, USA). The BioAnalyzer 2200 (Agilent, Santa Clara, CA, USA) with High-Sensitivity DNA Chips was used to verify fragment length distributions. After pooling of the library, quantification was conducted with the Qubit II Flurometer and the double-stranded DNA (dsDNA) High-Sensitivity Assay Kit (ThermoFisher Scientific, Life Technologies, Carlsbad, CA, USA). For the preparation of the final library, we used the 5% PhiXv3 control library for Illumina sequencing runs. Sequencing was performed in-house on the Miseq platform (500 cycles v2), on the sequencer located in the Department of Human Genetics of the University of Würzburg, Germany. Raw data are publicly deposited in the Sequence Read Archive (SRA) at the EBI (www.ebi.ac.uk/ena) with accession PRJEB38245.

### 2.3. Analysis

Forward and reverse reads were joined with fastq-join [39] and accepted only if longer than 250 bp. After that, quality filtering (Emax = 1, no ambiguous characters), dereplication (including filtering of singletons), denoising (including chimera removal), and clustering at 97% identity were performed with USEARCH v10 [40,41,42]. Finally, we assigned taxonomies using the RDP v16 reference database [43] using the same software. The resulting community and taxonomy tables are available as Supplementary Tables S2 and S3. The dataset was further analyzed in R 3.2.4. [44] using the packages phyloseq [45], ggplot2 [46], microbiome [47], MASS [48], and vegan [49]. Taxa assigned as chloroplasts, mitochondria, or unclassified were removed. After filtering, we computed the Shannon diversity for all samples, and an ANOVA test was conducted to compare these between healthy and deceased larvae. Data were relativized, and differences in community composition were visualized with Bray–Curtis-based non-metric multidimensional scaling (NMDS). Bacterial communities between larvae and respective community distance matrices for larvae and respective pollen provisions were compared with Mantel tests based on Pearson’s correlation to explore the degree of association between them [50]. Permutational multivariate analysis of variance (PERMANOVA) and betadisper were used to assess overlap and variability between and within sample groups. To find co-occurrences between bacterial taxa in deceased larvae, we used the program SparCC [51] for pairwise correlations with 1000 bootstrap replicates to calculate pseudo *p*-values. We only considered correlations with coefficients greater or less than 0.3 or −0.3, respectively, together with pseudo *p*-values less than 0.001, as relevant. For testing if treatment of the pollen influenced the larval bacterial microbiome significantly, we performed a linear discriminant (LD) analysis with treatment type set as the discriminative class.

## 3. Results

### 3.1. Bacterial Alpha Diversity and Composition of O. bicornis Nest Chambers

**Figure 1:**
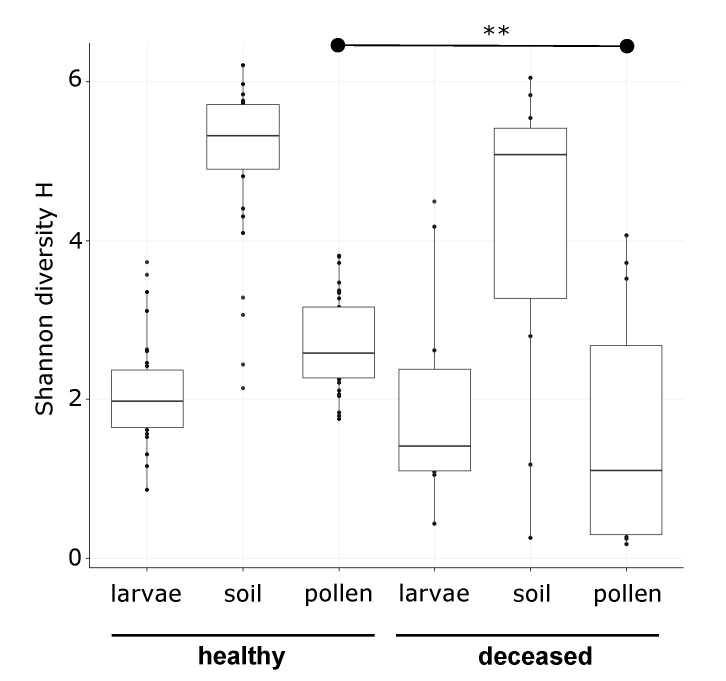
Shannon diversity of bacterial taxa found in larvae, pollen and soil from 31 nest chambers with healthy *Osmia bicornis* larvae and 12 nest chambers with deceased *O. bicornis* larvae.

We examined the larvae, pollen provisions, and soil from 43 *O. bicornis* nests by 16S rRNA gene metabarcoding. One pollen sample returned fewer than 1000 filtered reads and was excluded from downstream analysis. For the rest of the samples, sequencing generated a range of 1008 to 15,843 filtered reads (average: 4372.8 reads) for larvae, 1006 to 14,664 reads (average: 3538.3) for pollen, and 1726 to 29,697 reads (average: 10,748.3) for soil. Shannon alpha-diversity tended to be lower for deceased larvae, their nest material, and their pollen provisions (Figure 1), although the difference between deceased and healthy groups was only statistically significant for pollen provisions (ANOVA, F = 11.14, *p* < 0.01 **, µ_healthy_ = 2.7 and µ_deceased_ = 1.6), but not for larvae (ANOVA, F = 0.19, *p* = 0.664, µ_healthy_ = 2.1 and µ_deceased_ = 1.9) or for soil (ANOVA, F = 3.31, *p* = 0.076, µ_healthy_ = 5.0 and µ_deceased_ = 4.2).

**Figure 2:**
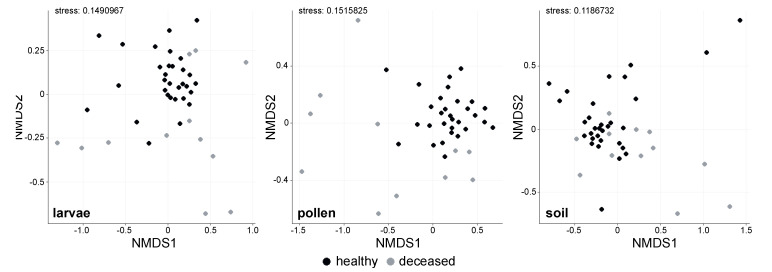
Bray–Curtis-based non-metric multidimensional scaling (NMDS) ordinations of larvae, pollen, and soil pellets acquired from *O. bicornis* nest chambers, where 31 contained healthy larvae and 12 contained deceased larvae.

NMDS ordination showed differences in the bacterial microbiome structure between the two groups in each investigated material (Figure 2). Bacterial microbiome differences between healthy and deceased larvae and their pollen provisions were both statistically significant (PERMANOVA, *R^2^* = 0.214, *p* < 0.001 *** and *R^2^* = 0.167, *p* < 0.001 ***, respectively), while beta dispersity of both datasets was homogeneous for deceased and healthy larvae (*p* > 0.05) and their respective pollen provisions (*p* > 0.05). Differences in soil bacterial microbiomes between the two groups were weak in comparison, but also significant (PERMANOVA, *R^2^* = 0.04, *p* < 0.05 *, betadisper *p* > 0.05).

**Figure 3:**
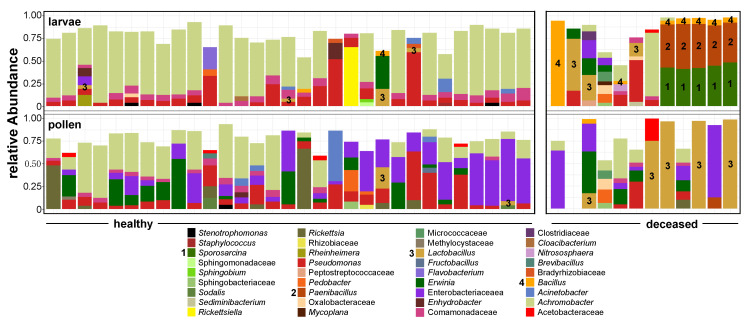
Bacterial composition in larvae (**top**) and pollen (**bottom**) samples acquired from 43 *O. bicornis* nest chambers belonging to different nests. Of these, 31 nest chambers contained healthy individuals (**starting left**) and 12 contained deceased individuals. Bacterial genera of special interest and further discussed in the text are referred to with numbers. Taxa are agglomerated up to genus level (family level if not further classifiable). Taxa contributing less than 5% to the dataset are not represented in the legend and bars.

The bacterial communities in healthy larvae were more homogeneous (beta dispersity: 0.29) than the ones in deceased larvae (beta dispersity: 0.56). The comparison between bacterial communities showed a compositional rise of mostly aerobic Firmicutes at the expense of Proteobacteria in deceased larvae and their pollen provisions. The dominant phylum in the bacterial communities of healthy larvae was Proteobacteria (88.43%), while, in the deceased larvae, Firmicutes constituted 59.67% of the community. This was mostly due to an increase in the genera *Bacillus* (mean relative abundance: 10.8%), *Paenibacillus* (mean relative abundance: 19.8%), and *Sporosarcina* (mean relative abundance: 18.2%) (Figure 3). These three genera also co-occurred significantly together (all cor >0.35, bootstrapped pseudo-*p* < 0.001 ***). The dominant *Paenibacillus* was further taxonomically classifiable into to the *Paenibacillus pabuli*/*amylolyticus*/*xylanexedens* phylogenetic cluster [52] with 100% sequence identity given the short investigated marker V4 16S rRNA gene. The bacterial communities in the respective pollen provisions were slightly more homogeneous between samples for healthy larvae (beta dispersity: 0.48) than for deceased larvae (beta dispersity: 0.57). Moreover, there was also a rise of Firmicutes (47.60% in provisions of deceased larvae) at the expense of Proteobacteria (86.58% in provisions of healthy larvae). This was almost entirely due to the abundance of lactobacilli in the community (Figure 3). At the same time pollen of healthy larvae showed low or undetectable levels of lactobacilli.

The bacterial composition datasets between larvae and their respective pollen provisions were correlated with Mantel tests and returned statistically significant associations. The matrix correlation value between the bacterial compositions of healthy larvae and their provisions was *r* = 0.30 (*p* < 0.05 *), and the respective value for deceased larvae and their provisions was *r* = 0.41 (*p* < 0.05 *).

The bacterial communities in soil were much more diverse (Figure 1) and, thus, their composition was constituted by more taxa with lower relative abundances. The most prevalent taxa were from the genus *Bacillus* (Firmicutes) (10.33% and 10.57% for nests with healthy and deceased larvae, respectively), the family of Gaiellaceae (Actinobacteria) (5.40% and 5.46% for nests with healthy and deceased larvae, respectively), and the genus *Achromobacter* (Proteobacteria) (5.95% and 2.30% for nests with healthy and deceased larvae, respectively).

### 3.2. *O. bicornis* Larvae Provisioning Experiment

We examined 31 larvae, which were reared under different provisioning settings. Larvae which fed on untreated pollen returned 1017 to 12,490 filtered reads (average: 5261.3), and larvae which fed on pollen that was previously inoculated with a bacterial solution (*Bacillus* sp. strain) returned 1090 to 3736 filtered reads (average: 2344.3). Interestingly, larvae which fed on sterile pollen only (group B) returned a low count of filtered reads (40 to 726, average: 313) and were therefore excluded from any downstream analysis. This indicates that larvae contained only a low bacterial density in comparison with the other individuals, although we did not perform quantitative analyses. The few remaining reads observed indicate that the individuals were not fully gnotobiotic; however, despite a depletion, some remaining bacteria were still present. In addition to larval samples of the three treatments, the original mixed pollen sample used to feed the larvae was also examined as a day zero pollen control (1643 filtered reads).

**Figure 4:**
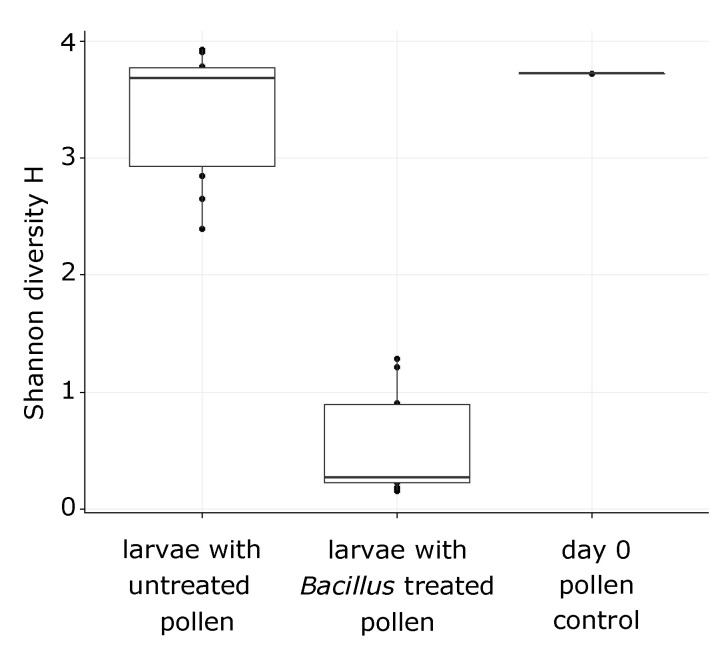
Shannon diversity of bacterial taxa found in (left) 11 larvae which fed on untreated pollens, (middle) 10 larvae which fed on sterilized pollen which was inoculated with the *Bacillus* sp. strain, and (right) one pollen sample at day zero of the experiments and then used to feed the larvae.

**Figure 5:**
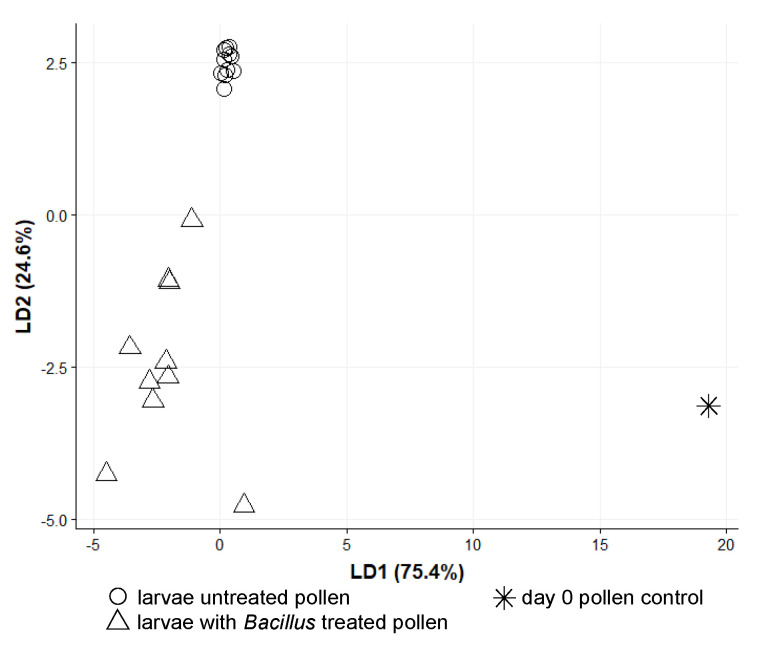
The two linear discriminants (LD1 and LD2) resulting from the linear discriminant analysis with (**a**) the original pollen sample used for feeding, (**b**) 11 larvae which fed on the aforementioned pollen sample, and (**c**) 10 larvae which fed on sterile pollen which was inoculated with one *Bacillus* sp. strain.

**Figure 6:**
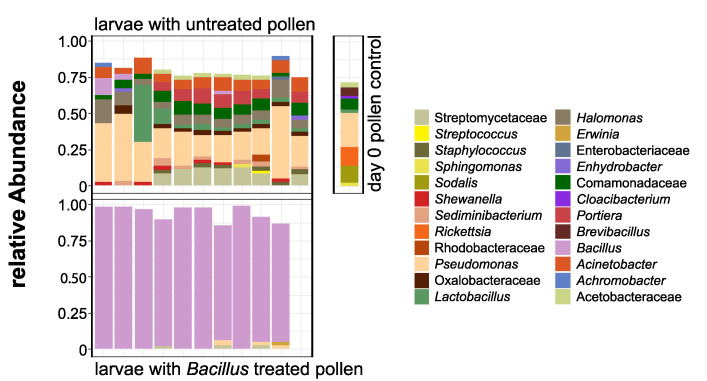
Bacterial composition of larvae which fed for five days on untreated pollen taken from one *O. bicornis* nest (**top left**), of the original fed pollen day 0 control (**top right**), and of larvae which fed for up to five days on pollen inoculated with one *Bacillus* sp. strain (**bottom**). Agglomerated up to genus level or family level if not better classifiable.

The bacterial alpha diversity of the groups A and C of larvae is shown on Figure 4. Multivariate dispersions within each group (A: provisions untreated, C: provisions sterilized and inoculated with the isolated *Bacillus* strain) were heterogeneous (dispersity values: 0.29751 and 0.06692, respectively, permutation test for homogeneity *p* < 0.001 ***). The type of treatment was successful as a discriminant factor for the bacterial communities found in manipulated larvae in the linear discriminant analysis (Figure 5), albeit mostly by the second LD axis which explained 24.6% of variance. The bacterial composition of the treated group C of larvae was entirely characterized by *Bacillus* sp. (Figure 6). Since we did not work with gnotobiotic larvae, we assume that bacteria already present were, therefore, replaced by this strain over the time of the experiment. These results together confirm that the larval microbiome was strongly changed by the inoculation and uptake of the bacterium. Moreover, this group C showed high mortality on day 4 after inoculation (six out of 10 larvae), while all individuals of groups A (untreated) and B (sterilized) survived until day 5.

## 4. Discussion

In this study, we firstly investigated the microbiomes of healthy and deceased red mason bees, *Osmia bicornis*, in the larvae themselves and their nest environment, i.e., pollen provisions and soil chamber separators. Altogether, we detected changes in microbiome diversity, variability, and composition, and we found specific bacteria that dominated the community in deceased larvae, while being undetectable in most healthy individuals or in low abundances.

As previously shown, the natural nest microbiome of *O. bicornis* is composed of a community of high bacterial diversity with Proteobacteria being the most prevalent phylum [19,23], which might to some degree originate from flowers [31,53]. The bacterial alpha diversity in healthy larvae was lower than the respective diversity in nest materials. Yet, the association correlation of larval and pollen bacterial microbiome was significant, indicating that the larval bacterial microbiome was linked to that of the provided pollen, as previously suggested [23]. Moreover, bacterial communities were fairly homogeneous between healthy larvae, which might be due to some degree of filtering regarding environmental bacteria, even if not to the extent as observed for honey bees [30,54]. For deceased individuals, the bacterial alpha diversity was lower than for healthy individuals in all three sample types. Nevertheless, this difference was significant only in the case of the pollen. The bacterial composition, however, was significantly distinct between the two groups and specifically for larvae and their respective pollen provisions. The health state of individuals can, thus, be reflected in or interact with the overall diversity of bacteria occurring in larvae and the nest. Furthermore, we found highly variable bacterial community compositions in deceased individuals and pollen provisions, but not the soil. Uncharacteristic bacteria might be able to directly harm larvae and, thus, disrupt the natural microbiome structure [55], indirectly generating a harmful environment or diet that supports a different and/or erratic bacterial community [56], or they might just thrive in this changed environment. The gut microbiome of living larvae acquires nutrients from the ingested pollen substrate, whereas, in deceased, larvae the primary resource was likely necrotic biomass, independent of whether they were causative pathogens or not. This, however, does not account entirely for the bacterial turnover observed in pollen provisions, where effects on diversity and community structure were strongest. In addition, other identities of bacteria dominated pollen provisions than seen in the respective deceased larvae, and the overall overlap of communities between pollen and larvae was lower than for healthy individuals. The observed link between the two communities seems to discontinue for dominant taxa after larval death with a consequential clearer separation.

Detailed screening for the deceased larvae revealed three bacterial taxa with dominant presence assigned to the genera *Paenibacillus*, *Sporosarcina*, and *Bacillus*. *Paenibacillus* is a notorious genus in host–microbe interactions, with a wide range from beneficial to pathogenic associations [57]. More specifically, *P. larvae* is considered to be the cause of acute mortal intestinal larval infections in honey bees, also known as the American foulbrood [11]. *P. alvei* is a saprophytic, aerobic bacterium which does not grow in healthy bee larvae, but can establish in diseased honey bee colonies in larval remains [58]. On the other hand, other members of the genus are associated with beneficial functions in solitary and stingless bee nests [52,59], protecting them from molding and other fungal threats. The here identified *Paenibacillus* was further taxonomically closely related to *P. pabuli, P. amylolyticus*, and *P. xylanexedens*, which are usually considered to be environmental, but are associated with co-infections with other bacteria in vertebrates [57,60]. They may have also established here as opportunistic secondary invaders in the deceased larvae, given their rich biochemical and particularly catabolic capacity, as is the case for *P. alvei* [57,58]. *Sporosarcina*, on the other hand, is less characterized as insect-related. Usually, it is considered environmental, although strains of the genus were identified for *Galleria mellonella* larvae and *Triatoma infestans*, which were infected with nematodes or trypanosomes, respectively [61,62]. The third dominating bacteria in larvae, *Bacillus* spp., form a wide and diverse bacterial group with members known as toxin-exerting and lethal for insect larvae and pupae [63]. Our microbiome network analyses showed that the three decease-associated taxa tended to co-occur together and were all negatively correlated with bacterial taxa representing the microbiome of healthy larvae. Similar networks were proposed to indicate candidate taxa for a number of desirable or undesirable outcomes like the presence or absence of specific infections [64]. From the microbiome alone, it is unclear whether any of these bacteria are the causative pathogens, secondary invaders, or degrading opportunists; therefore, we later performed experimental verification with the most likely pathogen candidate, i.e., *Bacillus* spp.

*Lactobacillus* spp. were prevalent in several pollen samples from the nests of deceased larvae, in contrast to pollen provisions of healthy larvae, where the ratio of the genus was very low. In previous studies, lactobacilli were found in the pollen provisions of the solitary bee genus *Megachile* and the species *Osmia caerulescens* [22,23,65], while, at the same time, they were mostly absent from *Osmia bicornis* nests and pollen provisions [19,22,23]. Lactobacilli are associated with key roles in pollen fermentation and defense against bacterial pathogens [66], possibly by secreting antimicrobial substances [67]. In our case, the lactobacilli found in high abundances for the deceased larvae might have originated from other bees via flowers [30,32,65,68,69]. They might then thrive on the large amounts of available organic pollen resources in the absence of an *Osmia bicornis* larvae and its microbial associates, which could both compete with and suppress *Lactobacillus* in the nest [52,59,70].

The soil used to separate individual nest chambers showed the highest bacterial diversity of all investigated materials, as expected [19]. Furthermore, the most dominant bacterial taxa, as well as the overall composition, were similar between healthy nests and deceased larvae. This indicates that the soil can sustain its natural microbiome in the bee nest independent of the health of the larvae. Therefore, apart from protection against intruders [9], its use as a nest construction element could also provide protection against pathogen spillover between the chambers of individual larvae.

In addition to general community assessments, we also conducted experimental provisioning assays with *O. bicornis* larvae to test further whether larvae take up bacteria, particularly pathogens, with their food. Bacterial diversity and composition for larvae that fed on untreated pollen was consistent with that of the original pollen provision itself, although with slightly higher diversity than field samples. This was probably due to pooling pollens from different nest cells. We can, thus, exclude that rearing larvae in the artificial environment itself had a major impact on bacterial diversity and composition. On the other hand, we observed less variability between microbiomes as a result of this controlled environment and a standardized pollen provision. The consistent overlap with pollen bacteria and reduced variability corroborates that the nest environment (particularly the pollen) is a major driver of larval microbiome community composition. Unfortunately, we were not able to assess the bacterial composition of larvae which fed on sterilized pollen for five days without inoculation. Given that, we were not able to make a direct compositional comparison between the two sterilized groups. Moreover, it is not fully confirmed that a combined chemical and UV treatment affected all bacteria, including potentially oxytetracycline-resistant environmental bacteria, and we cannot fully exclude that fungi surviving the sterilization procedure might still have had an influence on the microbiome structure. Nonetheless, the result that bacterial sequencing failed in all individuals hints at a depleted microbiome. Bacterial density in the gut was, therefore, most likely reduced without constant influx from the pollen provisions. Backward inoculation from larvae to pollen provisions seems also of minor importance as such a feedback loop would have maintained associations. A longer-term experimental study found that fitness of *Osmia ribifloris* decreased when the microbiome of the pollen provisions was reduced in density [31]. Depletion of a pollen microbiome might result in lack of essential functions for development, including pollen fermentation, spoilage inhibition, and pathogen defense (outside or within the larvae), or directly as a microbial food source [71]. Given that the complexity and volume of the gut both increase during transition from larvae to pupae for megachilid bees [72], influx of pollen bacteria might not be of equal significance at different stages of development.

Bacterial diversity found within larvae which fed on sterilized and *Bacillus*-inoculated pollen was low and mainly reduced to this strain. The consistent success of sequencing in these samples indicates that bacterial density increased in comparison with the samples which were only sterilized. This emphasizes that larvae and pollen provisions are in a rapid exchange of bacteria, and the food microbiome has a very strong impact on the larval microbiome. It was recently shown that solitary bee larvae can also acquire plant pathogens from their diet [73], and our results indicate that a potential bee pathogen can also be taken up this way. Lack of a pollen microbiome alone did not lead to higher mortality in our study; however, adding *Bacillus* sp. to this depleted microbiome resulted in acute mortality. This might be due to direct pathogenic capabilities of this bacterium, either as a transient or established gut component, or via indirect consequences of a disadvantageously composed and deranged gut community harming larval development, which are both factors to be further studied. Thus, ingesting a harmful bacterium had more severe consequences in our study than ingesting no microbes at all. However, the benefit of a diverse and dense pollen microbiome on bee fitness was shown previously [31] and might play a particular role in the long run by covering nutritional needs and repressing harmful bacteria. Overall, these results show that solitary bees are susceptible to hazardous bacteria (here the isolated *Bacillus*) imported through diet, and they contribute, together with other recent studies [31,71], to a general understanding that a supportive microbiome of pollen provisions is important for larval development.

## 5. Conclusions

In this study, we identified strong changes in bacterial microbiome diversity, composition, and variability when comparing healthy with deceased red mason bee larvae and their pollen provisions. This was mostly represented in a shift from a diversity of Proteobacteria, likely originating from flowers, to only four dominating Firmicute taxa. Inoculation of sterilized pollen with one of these strains led to a larval microbiome dominated by this bacterium and acute mortality, either directly via pathogenicity or passively via a changed gut community harming larval development. In addition, bacterial communities of individual pollen provisions and the corresponding larvae correlated, both in field and in experimental samples, and larvae supplied with sterilized pollen provisions yielded no identifiable bacterial gut community. These various insights support that the pollen provision microbiome has a strong and rapid influence on larval microbiomes. This makes solitary bees susceptible to passively introduced hazardous bacteria. On the other hand, the soil separating individual nest chambers was not affected by decease-related bacteria, and it might serve as a barrier to pathogen spread between individual larvae. All of this underlines the need for further investigation of microbial symbionts in the pollen, nest, and gut, as well as pathogens in solitary bees, to understand their impact on bee population dynamics and the importance of their natural microbiome for controlling pathogenic bacteria.

## Supplementary Materials

The following are available online at https://www.mdpi.com/2075-4450/11/6/373/s1, Table S1: Sample metadata, Table S2: OTU table, Table S3: Taxonomic classifications.
